# Assessing peripheral inflammation at single‐cell resolution in DS people at different stages of AD

**DOI:** 10.1002/alz70855_102800

**Published:** 2025-12-23

**Authors:** Natalia Valle‐Tamayo, Esther Álvarez‐Sánchez, Laia Muñoz, Joaquim Aumatell Escabias, Maria Carmona‐Iragui, Laura Videla, Bessy Benejam, Mateus Rozalem Aranha, Alexandre Bejanin, Daniel Alcolea, Juan Fortea, Oriol Dols‐Icardo

**Affiliations:** ^1^ Sant Pau Memory Unit, Hospital de la Santa Creu i Sant Pau, Institut de Recerca Sant Pau ‐ Universitat Autònoma de Barcelona, Barcelona, Spain; ^2^ CIBERNED, Network Center for Biomedical Research in Neurodegenerative Diseases, National Institute of Health Carlos III, Madrid, Spain

## Abstract

**Background:**

Neuroinflammation plays a major role in neurodegenerative diseases, including Alzheimer's disease (AD). In this context, accumulating evidence suggests that systemic inflammation and the infiltration of immune cells into the brain contribute to disease progression. Individuals with Down syndrome (DS) have a lifetime risk of up to 90% for developing Alzheimer's disease (DSAD), making them one of the largest populations with genetically determined AD. However, no study has investigated the role of the peripheral immune system in individuals with DS along the AD continuum at single‐cell resolution.

**Objective:**

We aimed to characterize peripheral blood mononuclear cells (PBMCs) from individuals with DS across different stages of AD, as well as from cognitively unimpaired healthy controls, using single‐cell RNA sequencing (scRNA‐seq). We correlated our in‐depth immune profiling with the levels of core AD biomarkers (Aβ1‐42, Aβ1‐40, pTau181, and NfL) measured using the Simoa platform.

**Methods:**

We included 15 cognitively unimpaired healthy controls from the SPIN cohort and 46 individuals with DS (22 with preclinical AD, 11 with prodromal AD and 13 with AD dementia) selected from the DABNI cohort. PBMCs were isolated using Ficoll density gradient centrifugation and cryopreserved until library preparation for scRNAseq and T‐cell receptor sequencing.

**Results:**

We anticipate identifying alterations in immune cell subpopulations and gene expression, as well as changes in cell‐cell communication patterns and a signature of T‐cell clonal expansion associated with progression to AD dementia and the presence of AD pathology, as determined using surrogate biomarkers.

**Conclusions:**

This is the largest study to date integrating high‐throughput single‐cell technologies with multimodal biomarker data in DS, providing key insights into the role of the peripheral immune system across AD stages and the presence of AD pathological hallmarks, particularly in the unique DS population.

